# Supplementary figures and images for: Performance evaluation of GenXplore q4800: a novel open-platform automated nucleic acid testing system
Source: Microbiol Spectr. 2026 Jan 21;14(3):e03092-25. doi: 10.1128/spectrum.03092-25 (PMC12955438; doi:10.1128/spectrum.03092-25)

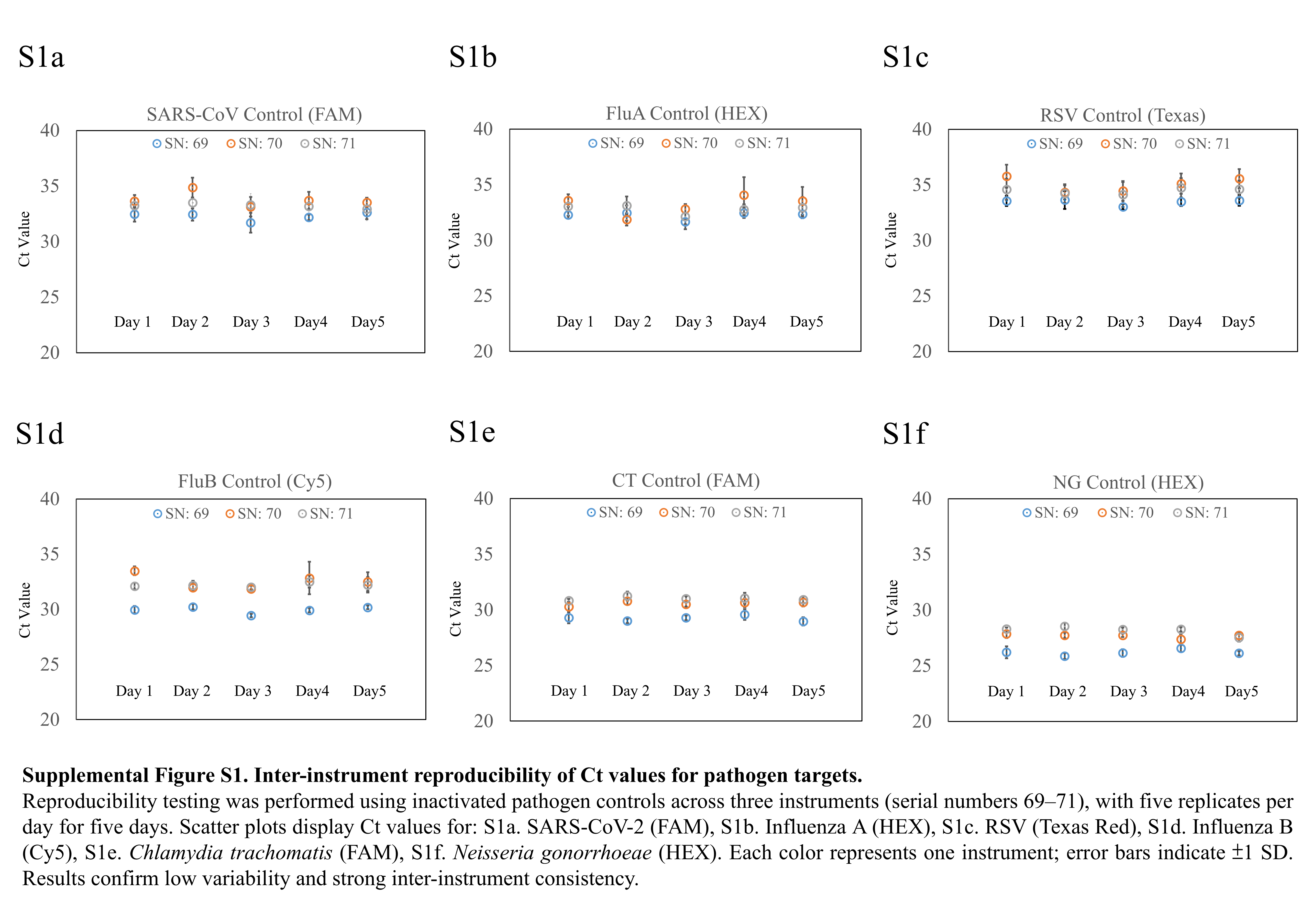

Supplement: Figure S1 — Inter-instrument reproducibility of Ct values for pathogen targets. [file spectrum.03092-25-s0001.tif]
